# Combining iCn3D and NextStrain to create a novel undergraduate research experience around SARS-CoV-2 variants and commercial antibodies

**DOI:** 10.3389/fgene.2023.1024063

**Published:** 2023-06-15

**Authors:** Sandra G. Porter, Todd M. Smith

**Affiliations:** ^1^ Digital World Biology LLC, Seattle, WA, United States; ^2^ Shoreline Community College, Biotechnology Program, Shoreline, WA, United States

**Keywords:** SARS-CoV-2, antibody, immunity, mutation, neutralizing, undergraduate research, iCn3D

## Abstract

Undergraduate research experiences are increasingly important in biology education with efforts underway to provide more projects by embedded them in a course. The shift to online learning at the beginning of the pandemic presented a challenge. How could biology instructors provide research experiences to students who were unable to attend in-person labs? During the 2021 ISMB (Intelligent Systems for Molecular Biology) iCn3D Hackathon–Collaborative Tools for Protein Analysis–we learned about new capabilities in iCn3D for analyzing the interactions between amino acids in the paratopes of antibodies with amino acids in the epitopes of antigens and predicting the effects of mutations on binding. Additionally, new sequence alignment tools in iCn3D support aligning protein sequences with sequences in structure models. We used these methods to create a new undergraduate research project, that students could perform online as part of a course, by combining the use of new features in iCn3D with analysis tools in NextStrain, and a data set of anti-SARS-CoV-2 antibodies. We present results from an example project to illustrate how students would investigate the likelihood of SARS-CoV-2 variants escaping from commercial antibodies and use chemical interaction data to support their hypotheses. We also demonstrate that online tools (iCn3D, NextStrain, and the NCBI databases) can be used to carry out the necessary steps and that this work satisfies the requirements for course-based undergraduate research. This project reinforces major concepts in undergraduate biology–evolution and the relationship between the sequence of a protein, its three-dimensional structure, and its function.

## 1 Introduction

For the past 4 years the annual Intelligent Systems in Molecular Biology (ISMB) meeting has hosted a hackathon related to developing and extending applications for iCn3D, a web-based software tool for visualizing and investigating molecular structures. The 2021 theme, Collaborative Tools for Protein Analysis, explored different ways to use iCn3D in research applications. By participating in the hackathon, we learned how to use new features in iCn3D to support undergraduate research.

Several studies have documented the contribution of undergraduate research opportunities to the likelihood that students will complete undergraduate degrees in STEM majors (Rodenbusch, et al., 2016; Cooper, et al., 2019). These data, combined with efforts to reform biology education (Woodin, Carter, and Fletcher, 2010) have prompted college biology programs to increase efforts to embed research opportunities into existing courses. Course-based undergraduate research opportunities (CUREs) allow greater numbers of students to participate in scientific research and develop the thinking skills that go along with such participation. The shutdowns driven by the COVID-19 pandemic highlighted the need for projects that could be completed remotely since many CUREs had to be suspended until students could return to a college lab.

Although most CUREs are implemented in laboratory courses, much scientific research takes place outside of a lab. Accordingly, we have been working to develop research projects that can be carried out by students working from home. During the ISMB hackathon, new features were added to iCn3D to support molecular interaction and mutation analyses and sequence alignments (Wang, et al., 2022). These new features, in addition to the knowledge we gained about using iCn3D, made us realize that analyzing the amino acid interactions between antibodies and SARS-CoV-2 spike proteins would be an ideal project for students working online.

To be successful, an on-line CURE requires the following components: a low-cost research tool that functions on multiple platforms (Windows, Mac OS, iOS), data, and an interesting research question that cannot be answered by a Google search. Ideally, the skills and knowledge gained through the project support the learning objectives for the course. The question of whether new variants of SARS-CoV-2 can escape antibody neutralization satisfies all these criteria.

Since December 2019, there have been over 766 million cases of SARS-CoV-2 infections and over 6.9 million deaths^1^. Visualization tools like NextStrain (Hadfield, et al., 2018) have allowed both scientists and students to follow SARS-CoV-2 evolution in real time as new variants like Alpha, Beta, Gamma, Delta, and Omicron, have emerged and spread (Tegally et al., 2022). All variants have acquired one or more mutations in the SARS-CoV-2 spike protein. Changes such as the D614G substitution increased viral transmissibility (Reviewed in Carabelli, et al., 2023). Other spike protein mutations improved the capacity for immune escape, especially in the case of Omicron (Carabelli, 2023).

That spike protein mutations impact the virus’ ability to spread is unsurprising given its critical role in infection. The spike protein is a trimer, located on the surface of SARS-CoV-2. The receptor binding domain (RBD) of the protein alternates between two states, up or down (Shang et al., 2020). In the up state, the RBD can contact the ACE2 protein, on the surface of a mammalian cell, and begin the process of infection. The RBD is also the principal target of neutralizing antibodies, many of which target epitopes that overlap with the ACE2 binding site (Jones et al., 2021; Cao et al., 2022; Liu et al., 2022; Wang et al., 2023). Mutations that change the antibody binding site can lead to immune escape by decreasing the affinity of antibodies for their target.

Over the course of the pandemic, several antibodies received emergency use authorizations (EUAs) from the FDA for treating or preventing infections with SARS-CoV-2 (Supplementary Table S1). Mutations in Omicron, and subvariants such as BQ and XBB, have rendered all previously authorized antibodies inactive (Wang et al., 2023), leading the FDA to withdraw authorizations^2^. The lack of effective antibodies for prophylaxis or treatment is problematic for people who are immunocompromised creating an unmet need for new treatments.

The combination of an important problem (the COVID-19 pandemic), existing data sets and freely available web-based analysis tools (iCn3D and NextStrain) make it possible for students to use current techniques to participate in research on anti-SARS-CoV-2 antibodies. To support this project, we developed a data set of structures from commercial antibodies and created protocols for obtaining spike protein sequences from SARS-Cov-2 variants, aligning those sequences to the spike proteins in the structures, and examining each amino acid change in iCn3D to predict the overall effect on antibody binding. In the CURE, students investigate the question of whether SARS-CoV-2 variants are likely to escape from an antibody. They identify mutations in the antibody binding site, compare the predicted chemical interactions between the antibody and amino acids in the original spike protein and those in the variant, and use the chemical interaction data to argue for or against the potential for immune escape.

## 2 Materials and methods

### 2.1 Antibody-spike protein structures

Several molecular structures are available for commercial antibodies complexed with the SARS-CoV-2 spike protein (Focosi, et al., 2022). Additional data were obtained from the Coronavirus Antibody Database (CovAbDab 2022), a resource that contained over 12,021 entries as of 21 March 2023 (Raybould, et al., 2021). We used these resources together with a literature review to assemble a data set of antibody names, drug names, and PDB IDs for ten commercial anti-SARS antibodies and their accompanying molecular structures (Supplementary Table S1) (Dejnirattisai et al., 2021; Dong et al., 2021; Focosi, et al., 2022; Hansen et al., 2020; Kim et al., 2021; Porter, et al., 2022; Tuccori, et al., 2020; Westendorf et al., 2022). In each case, the structures contain one or more antibodies bound to a SARS-CoV-2 spike protein. All data and instructional materials are available for instructors to download and use (Porter, et al., 2022).

Our example (PDB ID 7KMG) was obtained from the NCBI Structure database^3^. 7KMG contains the FAB portion of antibody LY-CoV555 bound to the RBD of the SARS-CoV-2 spike protein. LY-CoV555 was isolated from a convalescent COVID-19 patient and demonstrated high affinity binding to the spike protein, potent neutralizing activity, and the ability to outcompete binding to the ACE2 receptor (Jones et al., 2021). Marketed by Eli Lilly and Co. as Bamlanivimab, the antibody received an EUA in November 2020 and lost it in April 2021 due to an increased frequency of resistant variants^4^.

### 2.2 iCn3D

iCn3D is a web-based molecular modeling program first released in 2016 (Wang, et al., 2020) and freely available from the National Center for Biotechnology Information, 2022 ^5^. BLAST and Smith Waterman alignment algorithms have recently been embedded in iCn3D allowing protein sequences to be aligned to amino acid sequences within structure models. A new iCn3D mutation analysis feature predicts how molecular interactions between amino acids and other components of structure will change when an existing residue is replaced (Wang, et al., 2022). Eight kinds of molecular interactions: contacts (hydrophobic and Van der Waals interactions), hydrogen bonds, salt bridges (ionic interactions), halogen bonds, π cation interactions, and π stacking, are shown in iCn3D, both in 3D views and 2D diagrams. An advantage for instructors is that iCn3D users can share permanent, short, URLs for annotated structure models (Wang, et al., 2020), making it easier to evaluate student work. Anyone can paste a shareable URL in a web-browser and access an annotated molecular structure.

### 2.3 Implementation in the classroom

To implement the project with a class, an instructor would download the protocols (Porter et al., 2022), assign a different antibody to each student (or team of students), and provide them with the corresponding PDB ID for their molecular structure. Each structure contains one or more antibodies bound to the SARS-CoV-2 spike protein. Students retrieve their structure from the NCBI, open the file in iCn3D, and identifying the critical components–the spike protein, and the heavy and light chains of the antibody (or antibodies). The complete project includes five modules (Supplementary Table S2). Most recently this project was used in a Bioinformatics course at Shoreline Community College, WA (Fall 2022) and a microbiology course at Prairie State College, IL (Erica Lannan, personal communication).

### 2.4 Obtaining variant spike protein sequences from NextStrain

NextStrain is an online, interactive visualization platform, available at NextStrain.org, for analyzing the evolution of viral genomes (Hadfield, et al., 2018). NextStrain has two options for working with genome data from SARS-CoV-2, one instance uses data from GISAID, the other option uses open data from GenBank at the NCBI. The open data option (as of 05-27–2023) uses information from 2527 genomes, sampled between December 2019 and May 2023, and provides links to GenBank sequence records. This option makes it possible to retrieve amino sequences from spike protein variants. Several options exist for filtering data and displaying it in different ways.

To obtain a spike protein sequence from an emerging variant, we used a combination of NextStrain.org and Genbank at the NCBI. We went to NextStrain.org, found SARS-CoV-2 (COVID-19) and opened the “Latest global analysis–open data” instance. We used the Color By option and chose Emerging Lineage to highlight new lineages in the phylogeny for SARS-CoV-2. In May 2023, the emerging lineages, [BA.2.3.3.20 (9), BN.1 (96), CH.1.1 (54), XBB.1.9 (144), and XBB.2.3 (21)] represented 333 sequences, with the number of sequences for each shown in parenthesis.

**FIGURE 1:**
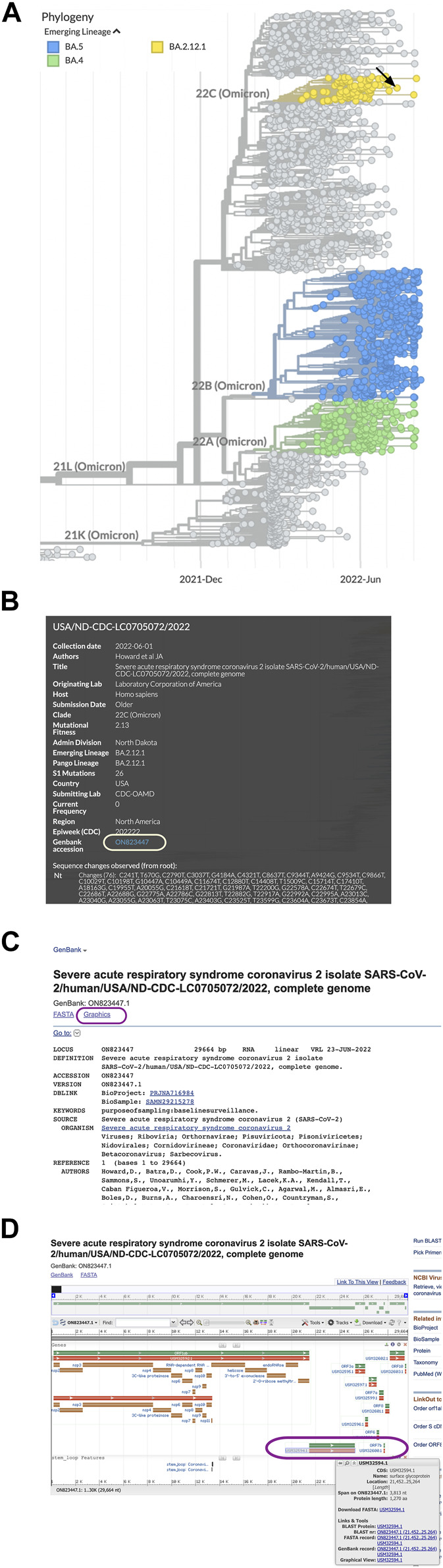
Obtaining sequence data for SARS-CoV-2 spike protein variants. **(A)** Emerging Lineages are highlighted in NextStrain phylogeny data for SARS-CoV-2. **(B)** Clicking a circle in the phylogeny opens a window with sample information and a link to the sequence in Genbank. **(C)** The Graphics link from the ON823447.1 GenBank record at the NCBI. **(D)** A map of SARS-CoV-2 genes and protein coding sequences. The gene (green) and spike protein coding sequence (orange) are in the lower right corner of the map (circled).

We chose to look at a sample in the BA.2.12.1 lineage (Omicron, isolated in 2022) by zooming in (Figure 1A) and clicking a dot to view the sample information (Figure 1B). The sample information contained a GenBank accession number (ON823447.1) that was linked to the corresponding genome sequence at the NCBI. We selected the link to access the NCBI genome record.

Once at the NCBI, we chose the Graphics link (Figure 1C) from the sequence record to view a genome map of SARS-CoV-2 with the locations of the coding sequences. To find the spike protein amino acid sequence, we held the cursor over the protein accession number (Figure 1D) to view a window with a link to the spike protein FASTA file and copy the accession number (USM32594.1).

### 2.5 Aligning a variant protein sequence to a spike protein sequence in a structure

**FIGURE 2:**
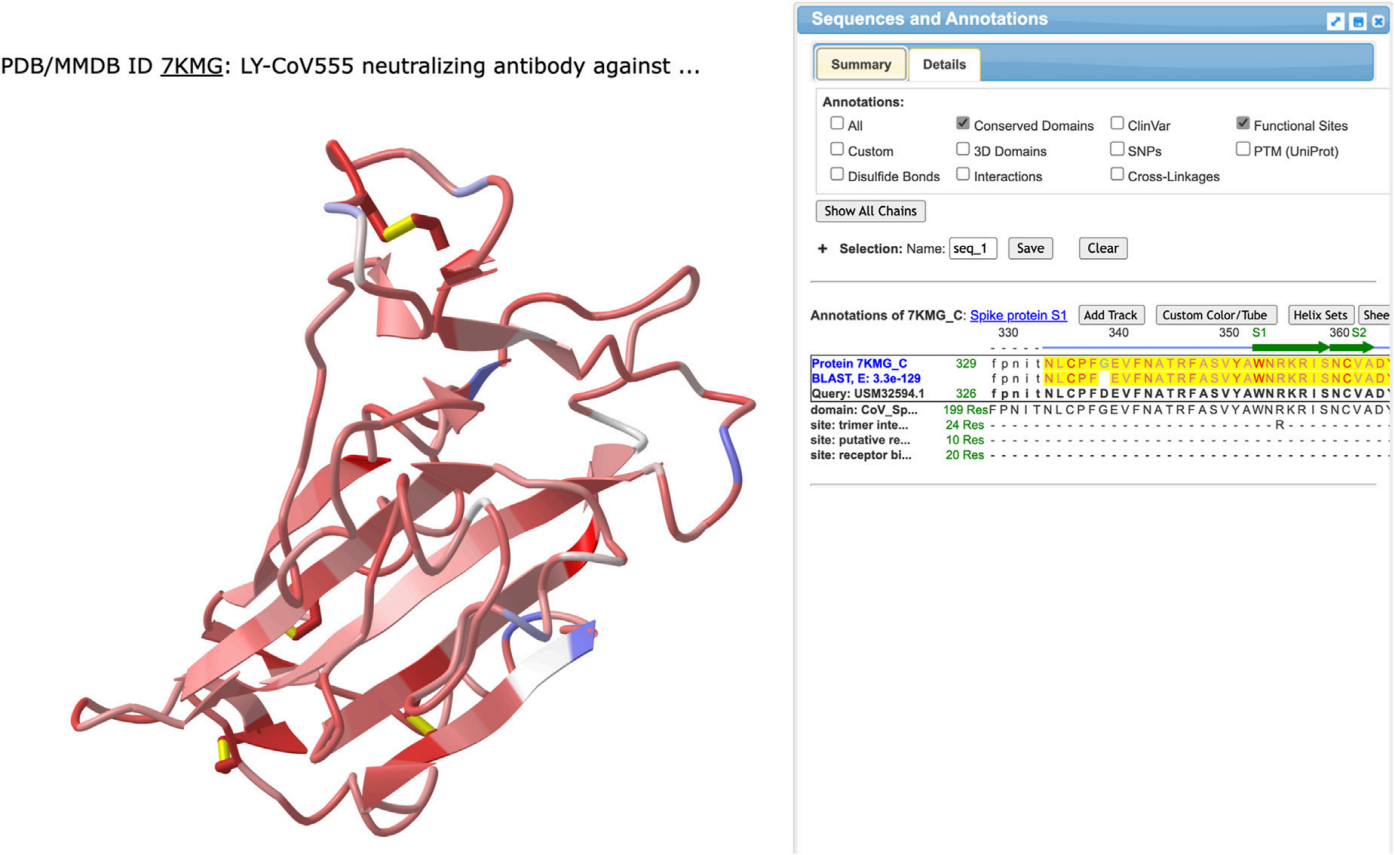
The results from using BLAST to align the variant spike protein sequence from USM32594.1 to the spike protein in 7KMG. The left side shows the 3D structure colored by identity. Identical or similar amino acids are colored red and pink, respectively. Positions where the amino acids are not conserved are colored blue. Aligned sequences are shown on the right with the 7KMG spike protein on top and the sequence from the BA.2.12.1 variant on the bottom. Identical residues (or a + for conserved residues) are shown in the middle row.

We searched the NCBI to find 7KMG in the structure database, identified the protein chains corresponding to the heavy and light chains of the antibody and the spike protein, then clicked the “full-featured 3D viewer” button to open the structure in iCn3D. We recorded the chain names for the antibody heavy chain, antibody light chain, and the spike protein for later use, then, opened the File menu, chose “Align” and “Sequence to Structure.” A window appeared where we pasted information about the protein sequence that would be aligned and the chain in the structure that it would be aligned with. The accession number for the variant protein sequence (USM32594.1) was pasted in the Sequence ID field and the chain name for the spike protein (7KMG_C) was entered in the NCBI protein accession field. Three alignment options are available: BLAST, Global Smith-Waterman, and Local Smith Waterman. We chose “Align with BLAST.” A 3D view of the spike protein sequence aligned to the 3D structure of the spike in 7KMG and the sequence is shown (Figure 2).

### 2.6 Adding annotations and identifying amino acid changes

**FIGURE 3:**
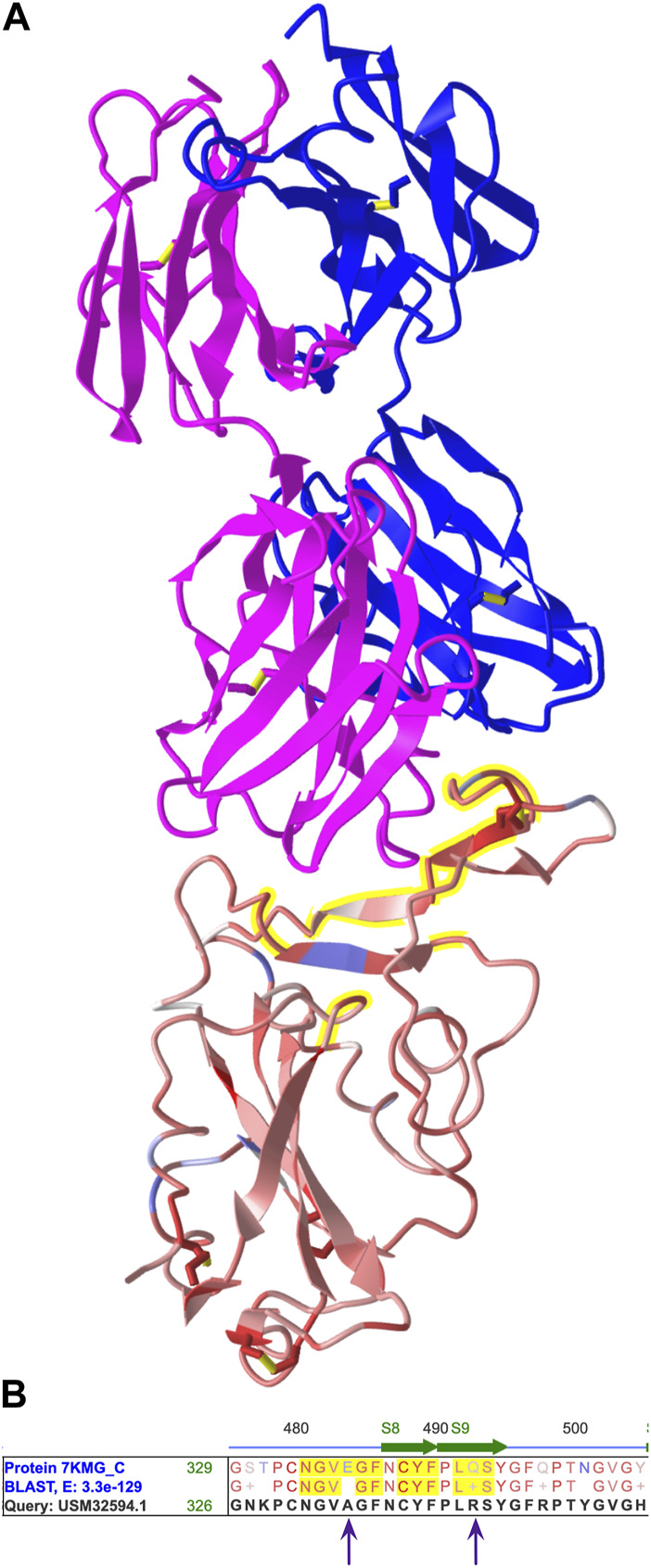
**(A)** The 3D structure from 7KMG. The heavy (pink) and light (blue) chains of the LY-CoV555 antibody are shown on top and the spike protein on the bottom. The antibody binding site is highlighted yellow. **(B)** A portion of the aligned amino acid sequences from the spike proteins in 7KMG and the BA.2.12.1 variant. Amino acids in the antibody binding are highlighted yellow. E484 and Q493 from 7KMG are replaced by alanine and arginine, respectively, in the variant. Shareable link: https://structure.ncbi.nlm.nih.gov/icn3d/share.html?MjythoEQ1qR1zzBw7.

We used annotations to identify amino acids in the antibody binding site and spot mutations. To annotate the antibody, we opened the Select menu, chose “Defined Sets”, and clicked the chain names for the heavy and light chains (7KMG_A and 7KMG_B). We opened the Select menu again, chose “Save Selection”, and changed the name to “antibody”. To show both the antibody and the spike protein, we opened the Select menu, chose Defined Sets, and selected both the antibody and the spike protein (7KMG_C). Then, we opened the View menu and chose “View Selection” to see both the antibody and the spike protein (Figure 3A).

Next, we annotated the antibody binding site. We opened the Select menu and chose the spike protein chain (7KMG_C). Then, we chose “by Distance.” For the first set, we selected the antibody, left the distance at 4 Å, chose the spike protein (7KMG_C) for the second set, and clicked Display. Amino acid residues within 4 Å of the antibody were highlighted yellow.

To identify mutations in the variant, we located the aligned spike protein sequences in the Sequences and Annotation viewer. We unchecked all the checked Annotation boxes to declutter the view and clicked the “Show All Chains” button to the locate the aligned spike proteins. As in the structure, amino acids in the antibody binding site were highlighted yellow. We scrolled through the aligned sequences and held the cursor over changed residues to obtain their position numbers. Two mutations, E484A and Q493R, were found in the antibody binding site (Figure 3B).

### 2.7 Mutation analysis

We evaluated the impact of spike protein mutations on antibody binding by using the mutation feature in iCn3D. The mutation option predicts chemical interactions between pairs of amino acids and provides information about interactions that would be shared and interactions that would change. We opened the Analysis menu, chose Mutation, and entered a mutation to investigate. It is possible to evaluate all the mutations at once but for students, it is more straightforward to interrogate the changes one at a time.

**FIGURE 4:**
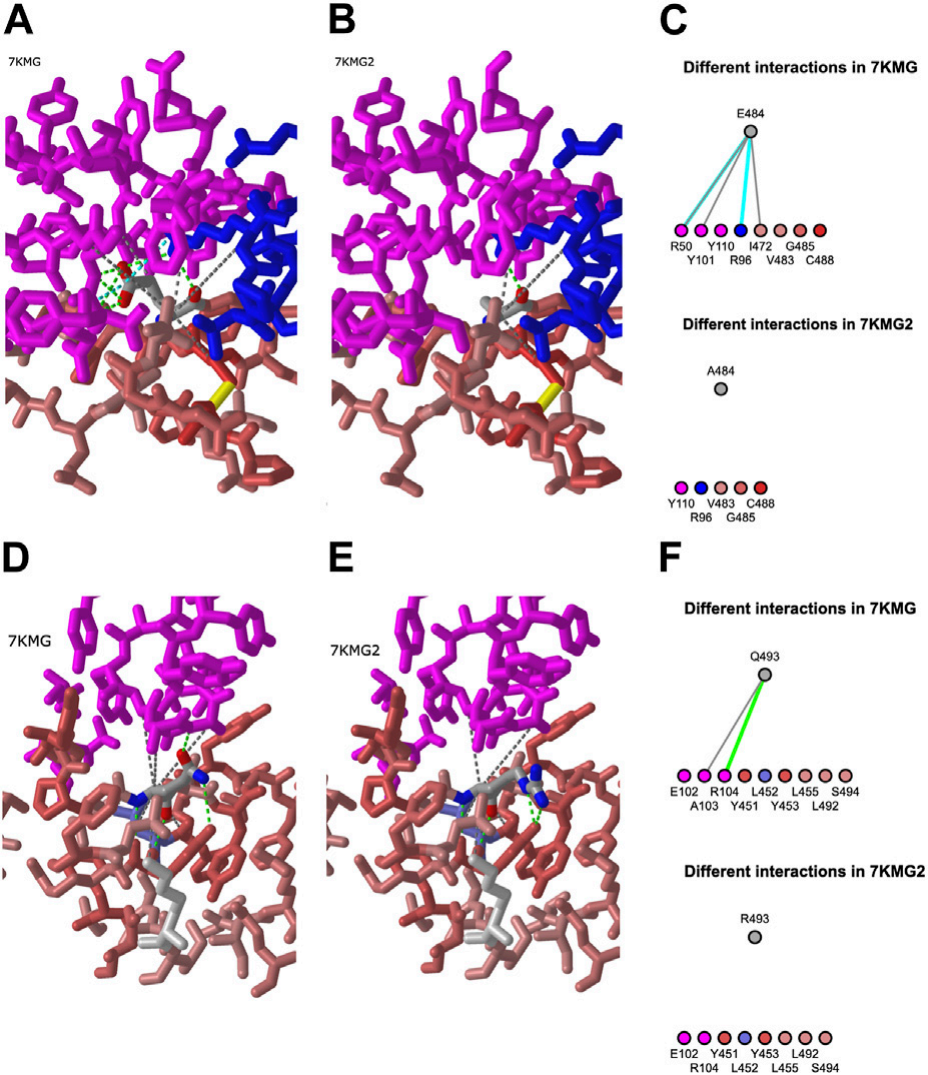
Mutation analysis in iCn3D. **(A)** Interactions between E484 (Glutamic acid) from the 7KMG spike protein (red-pink) and the Ly-CoV555 antibody heavy (pink) and light (blue) chains in the 3D structure. Green, cyan, and grey dotted lines represent hydrogen bonds, salt bridges, and contacts, respectively. Predicted chemical interactions between the A484 mutant and amino acids in LyCoV555 **(B)** and between the LyCoV555 antibody and E484 (top) or A484 (bottom) portrayed in 2D **(C)**, shareable link: https://structure.ncbi.nlm.nih.gov/icn3d/share.html?pJ1f1VSaZdxk9NPi8. **(D)** and **(E)** Chemical interactions and predicted chemical interactions for Q493 (left) and R493 (right). **(F)** Predicted changes in chemical interactions between the LyCoV555 antibody and Q493 (top) or R493 (bottom) portrayed in 2D, shareable link: https://structure.ncbi.nlm.nih.gov/icn3d/share.html?8hpoRtoxRyFMQrFz5.

To enter a mutation, the PD ID is listed first, followed by the chain name, the position, and the one letter code for the mutant residue. For E484A, this was 7KMG_C_484_A. We clicked the “Interactions” button to view the predicted change in both a 3D structure and a 2D diagram (Figure 4) and used the “A” key to alternate between the structures. We repeated the same process for Q493R. Both the 3D view and 2D diagrams were used for identifying interactions that might be retained in the mutant and those that would change.

## 3 Discussion

### 3.1 Analysis of LY-CoV555 binding and impact of mutations

In this example project, we identified two mutations (E484A and Q493R) in the LY-CoV555 antibody binding site for sequence USM32594.1, a spike protein from an Omicron sample in the BA.2.12.1 lineage. The 3D structure models (Figure 4) show results from using the mutation feature in iCn3D to predict how mutant amino acids will interact with other components in a structure. The E484A mutation replaces a glutamic acid at position 484 of the spike protein with an alanine. The mutation feature in iCn3D predicted that both E484 and A484 would form contacts with Y110 in the heavy chain and R96 in the light chain. In the case of different interactions, E484 has five interactions with the heavy chain: a hydrogen bond and contact with R50, a contact with Y101, and both a hydrogen bond and salt bridge with R96. The mutant, A484 is predicted to form a hydrogen bond with R96 in the light chain. Thus, four of the interactions between E484 and the antibody, one of which is a salt bridge (a strong interaction), would be lost with A484. From these results, we predicted that E484A would have a negative effect on antibody binding. This prediction is consistent with several publications demonstrating that mutations at E484, including E484A, allow immune escape (Starr et al., 2021; Cao, et al., 2022; Wang, et al., 2023).

In the case of Q493R, both glutamine and arginine are predicted to share contacts with E102 and R104. In terms of differences, Q493 contacts A103 and has a hydrogen bond with R104. Neither interaction would be present in the R493 mutant. With two more interactions for glutamine 493 than alanine, we predict that the Q493R mutation also disrupts binding. Again, this prediction is consistent with published work (Starr et al., 2021; Cao, et al., 2022; Wang, et al., 2023).

Since the E484A and Q493R mutations prevent Ly-CoV555 from binding to the variant spike protein, Bamlanvimab, the Ly-CoV555 antibody drug, no longer neutralizes the SARS-CoV-2 virus. As a result, the drug no longer protects people from SARS-CoV-2 infection nor can it be used to successfully treat people with COVID-19.

### 3.2 Student results

This project was piloted by students in a 5-week online bioinformatics course (Biol285) at Shoreline Community College during Fall 2021 and Fall 2022. Students in the course have a wide variety of backgrounds. Some are changing careers. Some have Bachelors’ degrees or advanced degrees from non-US institutions. Some are the first members of their family to attend college, and some went straight to college from high school.

We used this project with 13 students in our Fall 2022 course. Student evaluations noted the timeliness of the project and that they appreciated working on a current and important topic. All students were able to complete modules 1–4 (Supplementary Table S2) but only 7 out of 13 successfully completed module 5 (immune escape). Some of their challenges included using the wrong sequence, using the wrong structure, aligning their sequence to the wrong structure, misidentifying parts of a structure, ignoring some of the mutations, or incorrectly identifying the antibody binding site. iCn3D shareable URLs were critical for diagnosing where students went wrong. In the future, we will build in more checkpoints where students send shareable URLs to their instructors. Instructors will be able to use the shareable URLs to review student work and quickly determine when students are taking a wrong turn to help get them back on track.

### 3.3 Investigating SARS-CoV-2 immune escape as a CURE

This project meets the need for a computational undergraduate research project that can be embedded in a biology-related course. NextStrain, the NCBI databases, and iCn3D are free, easily accessed, and available to any student with a web browser and an internet connection, allowing the project to be implemented at a wide variety of educational institutions with few upfront costs. The process for using these tools can be applied to other pathogens allowing a quick response to the next pandemic.

CUREs require research questions that can be investigated by students in a limited time. Instructors require CUREs that discourage cheating and have results that can be assessed. This project meets those criteria. Every student (or team) is likely to research a unique combination of a sequence variant and an antibody. The evolution of SARS-CoV-2 continues to produce new spike protein variants (333 as of May 2023), making it unlikely that two students would choose the same variant from NextStrain and be assigned the same antibody by the instructor. Students may be able to search the literature and learn if mutations allow immune escape, but they are less likely to find detailed descriptions of the chemical interactions between a specific antibody and a mutant and be able to count the number and types of interactions that change. They would also be unlikely to find a shareable URL for their annotated structure model that they could hand in with their report. In summary, the requirement to investigate all the molecular interactions between amino acids in the antibody and those in the spike protein, combined with the large number of genome samples and combinations of antibodies with different variant proteins, make it unlikely that students will be able to solve their research problem using Google.

The global pandemic emergency has been declared over by the World Health Organization^6^ but the question of immune escape remains an important research problem. There is a still a need for drugs to prevent infection and drugs that can treat COVID-19 in immunocompromised patients. Companies and research laboratories are continuing to work on new antibody drugs (Liu et al., 2023). This project can expand beyond the original set of commercial antibodies as new antibody structures become available.

Carrying out this project helps students meet common biology learning goals. Unlike other molecular modeling programs, iCn3D makes the relationship between protein sequences, their structure, and their function explicit. The ability to identify molecular interactions and view them in both 2 and 3 dimensions reinforces the importance of chemical bonds and their contribution to protein interactions. Integrating genomics resources with molecular modeling is a multi-omics methodology that drives home the important relationship between sequence, structure, and function.

## Data Availability

Publicly available datasets were analyzed in this study. This data can be found here: https://qubeshub.org/publications/2913/1.

## Footnotes

1
https://covid19.who.int

2
https://www.fda.gov/drugs/emergency-preparedness-drugs/coronavirus-covid-19-drugs

3
https://www.ncbi.nlm.nih.gov/Structure/pdb/7KMG

4
https://www.fda.gov/news-events/press-announcements/coronavirus-covid-19-update-fda-revokes-emergency-use-authorization-monoclonal-antibody-bamlanivimab

5
https://www.ncbi.nlm.nih.gov/Structure/icn3d/full.html

6
https://news.un.org/en/story/2023/05/1136367
