# Supplementary material for: Combining iCn3D and NextStrain to create a novel undergraduate research experience around SARS-CoV-2 variants and commercial antibodies
Source: Front Genet. 2023 Jun 15;14:1024063. doi: 10.3389/fgene.2023.1024063 (PMC10311211; doi:10.3389/fgene.2023.1024063)
Supplement: Supplementary file 1 [file Table1.docx]

Supplementary Material

Combining iCn3D and NextStrain to create a novel undergraduate research experience around SARS-CoV-2 variants and commercial antibodies

Sandra G. Porter*, Todd M. Smith

*** Correspondence:** Corresponding Author: sandra@digitalworldbiology.com

# Supplementary Data

Table 1. Commercial drugs, antibodies, and corresponding structures

| **Drug** | **Antibody** | **PDB ID** | **Reference** |
| --- | --- | --- | --- |
| Bamlanivimab | LY-CoV555 | 7KMG | Jones, et al., 2021 |
| Etesevimab* | LY-CoV481 | 7KMI | Jones, et al., 2021 |
| Etesevimab | LY-CoV016 (CB6) | 7CO1 | Jones, et al., 2021 |
| REGN-COV* | Casirivimab (Regn10933) | 6XDG | Hansen, et al., 2020 |
| REGN-COV | Indevimab (Regn10987) | 6XDG | Hansen, et al., 2020 |
| Bebtelovimab | Bebtelovimab (LY-CoV1404) | 7MMO | Westendorf et al., 2022 |
| Evushield* | Cilgavimab (AZD1061) | 7L7E | Dong et al., 2021 |
| Evushield | Tixagevimab (AZD8895) | 7L7E | Dong et al., 2021 |
| Sotrovimab | S309 | 7BEP | Dejnirattisai et al., 2021 |
| Regkirona | Regdanvimab (CT-P59) | 7CM4 | Kim et al., 2021 |

*Three drugs were cocktails of two antibodies.

Table 2. Modules and research goals

| **Module** | **Research goals** |
| --- | --- |
| 1 | Compare structures with open and closed conformations of spike protein – recognize where ACE2 binds and identify the different conformations. |
| 2 | Find and align 3D structures to determine if the antibody blocks binding to ACE2.   1. Align the structures to see where protein might be located relative to another. 2. Identify different components of structures (antibody heavy and light chains, spike protein). 3. Create lifetime URLs. |
| 3 | Identify and annotate amino acids in the spike protein that contact the antibody. |
| 4 | Use NextStrain to find a variant, get the spike protein sequence from NCBI, and save the ID. |
| 5 | Evaluate the potential for immune escape.   1. Use BLAST in iCn3D to align the protein sequence from the variant to the spike protein sequence in the structure. 2. Annotate the antibody and the antibody binding site. 3. Identify mutations in the antibody binding site. 4. Look at each individual mutation and use the mutation tool in iCn3D to predict the changes in chemical bonds. 5. Predict whether the antibody will bind to the variant and use the predicted changes in chemical interactions to support your argument. |
